# Benzoate of Sodium in Throat Affections

**Published:** 1888-06

**Authors:** 


### *BENZOATE OF SODIUM IN THROAT AFFECTIONS.*

In the St. Louis *Courier of Medicine* for February, 1888, Dr. L. Boisliniere states that for the last two years he has been using benzoate of sodium in the treatment of various throat-troubles with very satisfactory results. He has employed it in acute follicular tonsillitis, acute erythema or œdema of the fauces, and in diphtheritic affections. Fifty-one cases of acute follicular tonsillitis were treated with the following formula:

℞ Benzoate of sodium, . . . . . . . . . . . ʒi-ʒiv.

Glycerin, . . . . . . . . . . . . . . .

Elixir of Calisaya bark, . . . . . . āā ℥i. M.

Sig.—One teaspoonful every one or two hours.

No local application was used. The author states that in the analysis of the last seventy-five cases treated, he found that the use of benzoate of sodium led to the relief of the disease in twelve to thirty-six hours; forty-one cases reporting well in twelve hours, thirty-one in twenty-four, and three in thirty-six hours, giving, therefore, an average of twenty hours the duration of each case. This drug likewise appeared to control the febrile elements in the disease, and may be given with impunity even to children, and is free from disagreeable effects. By acute erythema or œdematous sore throat, the author refers to that affection described as ordinary sore throat, caused by exposure to cold and wet, a trouble in which there is a redness, involving the tonsils, anterior and posterior pillars of the fauces, uvula, and pharynx, accompanied by œdema of the dependent parts, pain, and deglutition, slight fever, and general malaise. In these cases, benzoate of sodium, combined frequently with some mild astringent, appeared to abort the trouble and produce rapid relief. During epidemics of throat diphtheria, many of the exudative throat affections assume a diphtheroid appearance; thus, in acute tonsillitis, the discrete, inflamed follicles coalesce, and the exudation becomes inspissated and somewhat organized and membranous. This is with difficulty detached from the tonsils, and, when forcibly removed, leaves bleeding points. It very closely resembles diphtheria, and in many cases it is impossible to make a positive differential diagnosis, although the membrane is usually much whiter than in diphtheria. Benzoate of sodium, according to the author, usually cures such cases in two or three days, and thus assists in the diagnosis. In some cases, however, the membrane has not this white, flaky appearance, but is dark, often black in parts, yellowish, thick, and very tenacious. Four or six cases the author states were treated by the following prescription:

℞ Corrosive sublimate . . . . . . . . . . . . gr. ss.
Benzoate of sodium . . . . . . . . . . . . ℥ ss-℥ i.
Distilled glycerin, . . . . . . . . . . . .
Elixir of Calisaya bark . . . . . . . . . . āā ℥ ii. M.

Sig.—One teaspoonful every one or two hours.

In each of these cases, the membrane is said to have disappeared in from three to six days. Taking into consideration the fact that these cases were cured so rapidly, the author believes them to have been cases of acute tonsillitis modified by an existing epidemic of diphtheria. In true diphtheria, however, the author claims that benzoate of sodium will soften the membrane, control the fever, and materially shorten the duration of the disease.

